# Emerging functions of branched ubiquitin chains

**DOI:** 10.1038/s41421-020-00237-y

**Published:** 2021-01-26

**Authors:** Michael E. French, Chad F. Koehler, Tony Hunter

**Affiliations:** 1 Department of Biology, Grinnell College, Grinnell, IA 50112 USA; 2 Department of Chemistry & Biochemistry, Middlebury College, Middlebury, VT 05753 USA; 3 Molecular and Cell Biology Laboratory, Salk Institute for Biological Studies, La Jolla, CA 92037 USA

**Keywords:** Proteomics, Ubiquitylation, Proteasome

## Abstract

Ubiquitylation is a critical post-translational modification that controls a wide variety of processes in eukaryotes. Ubiquitin chains of different topologies are specialized for different cellular functions and control the stability, activity, interaction properties, and localization of many different proteins. Recent work has highlighted a role for branched ubiquitin chains in the regulation of cell signaling and protein degradation pathways. Similar to their unbranched counterparts, branched ubiquitin chains are remarkably diverse in terms of their chemical linkages, structures, and the biological information they transmit. In this review, we discuss emerging themes related to the architecture, synthesis, and functions of branched ubiquitin chains. We also describe methodologies that have recently been developed to identify and decode the functions of these branched polymers.

## Introduction

**Fig. 1:**
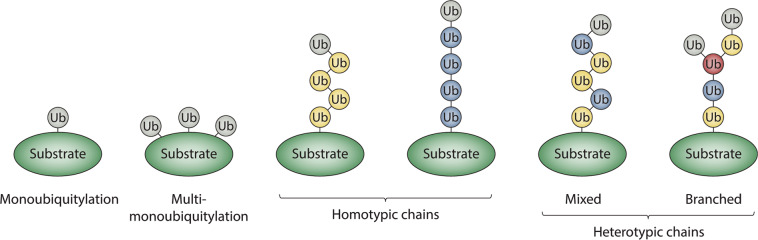
Classification of ubiquitin modifications. Protein substrates can be modified with ubiquitin monomers on one or more acceptor sites, referred to as monoubiquitylation or multi-monoubiquitylation, respectively. Alternatively, ubiquitin monomers can be joined to each other via isopeptide bonds to form chains of varying lengths, linkages, and structures. Homotypic chains are linked uniformly through the same acceptor site of ubiquitin (e.g., K48-linked chains), whereas heterotypic chains contain multiple types of linkages and can be further classified as either mixed or branched. Mixed chains consist of ubiquitin subunits that are modified on only a single acceptor site. Branched chains contain at least one ubiquitin subunit that is simultaneously modified on multiple acceptor sites. Ubiquitins modified on one acceptor site are colored in blue or yellow; the branch point ubiquitin is colored in red; unmodified or “terminal” ubiquitins are colored gray.

Ubiquitylation is an essential post-translational modification that controls the stability and functions of eukaryotic proteins through multiple mechanisms. Ubiquitylation regulates an assortment of processes that are of vital importance to eukaryotic cells, including cell division, differentiation, protein quality control, gene expression, DNA repair, protein trafficking, and signal transduction^1–5^. The ability of ubiquitin to act as a multifunctional signal is due to its propensity to form a variety of different structures that can be recognized by different types of effector proteins. Ubiquitin can be conjugated to substrates as a monomer on one or more sites (most frequently lysines), referred to as monoubiquitylation or multi-monoubiquitylation, respectively, or it can be polymerized to form a chain, in which the carboxy terminus of one ubiquitin monomer is linked to the ε-amino group of a lysine residue or the α-amino group of the N-terminal methionine of another ubiquitin monomer via an isopeptide bond (Fig. 1).

Ubiquitin chains can be classified into three different categories based on the types of linkages that connect adjacent ubiquitin monomers within the chain. Homotypic chains are linked uniformly through the same acceptor site of ubiquitin, whereas heterotypic chains are linked through multiple sites and can be further classified as either mixed or branched (Fig. 1). Mixed chains consist of more than one type of linkage, but each ubiquitin monomer within the chain is modified on only one acceptor site. By contrast, branched chains are comprised of one or more ubiquitin subunits that are simultaneously modified on at least two different acceptor sites. While the functions of homotypic chains are generally well-established, with, for example, K48-linked chains targeting proteins for degradation by the proteasome and M1- and K63-linked chains regulating vital processes such as DNA repair, NF-κB signaling, and autophagy^6–9^, the structures and functions of branched chains have started to emerge only recently. Similar in design to branched oligosaccharides on the cell surface, which can adopt a variety of different structures and have a crucial role in cell–cell adhesion, branched ubiquitin chains greatly increase the complexity of ubiquitylation signals and thus expand the types of biological information that can be transmitted by such signals. In this review, we discuss the most recent findings on branched ubiquitin chains, highlighting their specific architectures, mechanisms of synthesis, and proposed functions. We also describe some of the approaches and techniques that have recently been developed to detect and study the functions of these branched polymers.

## Architecture and synthesis of branched ubiquitin chains

**Fig. 2:**
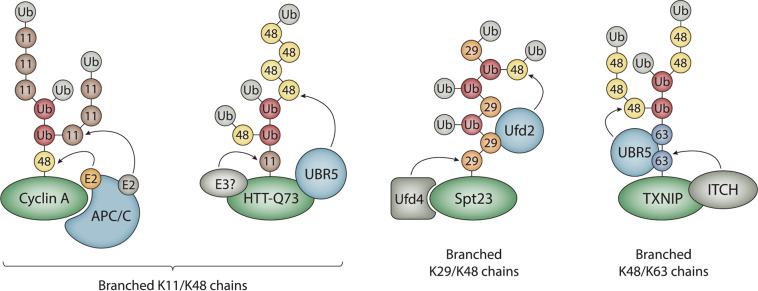
Architecture and synthesis of branched ubiquitin chains. The APC/C collaborates with two different E2s, UBE2C and UBE2S, to assemble branched K11/K48 chains on cyclin A and other mitotic substrates. UBR5 collaborates with an unknown K11-specific E3 to form branched K11/K48 chains of a different architecture on a pathological mutant version of the Huntingtin protein (HTT-Q73). UBR5 has also been reported to synthesize branched K11/K48 chains on newly synthesized misfolded polypeptides^11^. Spt23 and substrates of the ubiquitin fusion degradation pathway are modified with branched K29/K48 chains synthesized by Ufd4 and Ufd2. ITCH cooperates with UBR5 to assemble branched K48/K63 chains on the pro-apoptotic regulator TXNIP. Substrates are colored in green; chain branching E3s are colored in light blue; the color coding for ubiquitins is as described in Fig. 1.

There are many recent reports of branched ubiquitin chains, and these polymers differ from each other in terms of their length, linkage, and overall architectures (Fig. 2). Branched chains with clear physiological functions include those consisting of K11/K48, K29/K48, and K48/K63 linkages^10–15^. Branched K6/K11, K6/K48, K27/K29, and K29/K33 chains have been detected in vitro or in cells but currently have unidentified functions^16–18^. Evidence also exists for heterotypic M1/K63 and K11/K63 polymers^19–23^, although it is unclear if these chains are mixed, branched, or contain a combination of both chain types. The potential for a nearly limitless number of distinct structures exists because branched chains can be formed through unique combinations of acceptor sites and because branch points can be initiated at distal, proximal, or internal ubiquitins within the chain. In addition, branched chains with the same types of linkages can differ in their overall architectures depending on the order in which the linkages are synthesized (Fig. 2). For example, the APC/C forms branched K11/K48 chains by assembling K11 linkages on preformed K48-linked chains, whereas UBR5 forms branched K11/K48 chains by attaching K48 linkages to preformed K11-linked chains^10,11^.

The synthesis of all ubiquitylation signals, including branched ubiquitin chains, requires the sequential actions of at least three different types of enzymes: E1, E2, and E3^24,25^. E3 ubiquitin ligases, of which there are predicted to be ~600 in humans, are responsible for catalyzing the transfer of ubiquitin monomers to substrates and for building ubiquitin chains on substrates. E3s can be classified into several different categories based on the presence of conserved catalytic domains and the mechanism of ubiquitin transfer that they use. The really interesting new gene (RING) and U-box E3s promote the direct transfer of ubiquitin from the E2 to the substrate or substrate-conjugated ubiquitin molecule. In contrast, the homologous to E6AP C-terminus (HECT), RING-between-RING (RBR), and recently identified RING-Cys-Relay (RCR) E3s employ a two-step mechanism of ubiquitin transfer, in which ubiquitin is first transferred from the E2 to a cysteine residue in the active site of the E3 and then from the E3 to a substrate or substrate-conjugated ubiquitin^26–29^.

While several different mechanisms for chain formation and linkage site selection for homotypic polymers have been described^6,30^, the mechanisms that underlie the formation of branched polymers by the E3s that synthesize them are less clear. One common theme in the assembly of branched chains is collaboration between pairs of E3s with distinct linkage specificities (Fig. 2). For example, Ufd4 and Ufd2 collaborate with each other to synthesize branched K29/K48 chains on substrates of the ubiquitin fusion degradation (UFD) pathway in yeast^13^. Similarly, branched K48/K63 chains are produced by TRAF6 and HUWE1 during NF-κB signaling, and by the HECT E3s ITCH and UBR5 during the apoptotic response^14,15^. One likely function of collaboration between pairs of E3s with distinct chain linkage preferences is to spatially and/or temporally separate ubiquitylation marks with different consequences. The pro-apoptotic regulator TXNIP, for example, is first modified with non-proteolytic K63-linked chains by ITCH before UBR5 attaches K48 linkages to produce branched K48/K63 chains, resulting in the subsequent degradation of TXNIP by the proteasome^15^. The conversion of a non-degradative signal to a degradative mark could be an efficient means of regulating the activation and inactivation of signaling proteins that are controlled by ubiquitylation events.

Other mechanisms of branched-chain formation involve a single E3 that either recruits E2s with different linkage specificities or has the innate ability to synthesize chains of different linkages on its own. The APC/C, a multisubunit RING E3, cooperates with two different E2s, UBE2C and UBE2S, to form branched K11/K48 chains on substrates during mitosis^10,11^. In this case, UBE2C first attaches short chains containing mixed K11, K48, and K63 linkages to substrates of the APC/C, and the K11-specific E2, UBE2S, then adds multiple K11 linkages to these short chains, resulting in branched K11/K48 (and probably also branched K11/K63) polymers^10,31^. The HECT E3 WWP1 has been demonstrated to synthesize branched chains containing K48 and K63 linkages in the presence of a single E2, UBE2L3^32^, whereas UBE3C and the bacterial HECT-like E3 NleL have been reported to assemble branched K29/K48 and K6/K48 chains, respectively, also in the presence of a single E2^17,18,33^. The RBR E3 Parkin, which is often mutated in early-onset Parkinson’s disease, has recently been shown to synthesize branched K6/K48 chains^34^, consistent with previous reports that Parkin forms chains of complex topology including multiple linkages^35–37^.

Regardless of whether the formation of branched chains involves an individual E3 or a pair of collaborating E3s, the initiation of chain branching requires the selection of the appropriate branch point linkage and location. For branched chains formed by E3s that work together in pairs, the E3 that initiates branching must recognize an initial mark that contains a particular linkage that is distinct from the one it synthesizes. Ufd2, for example, recognizes K29-linked chains assembled by Ufd4 and initiates branching by adding multiple K48-linked ubiquitins to the chain. It does this by binding to K29 linkages through two loops present in the N-terminal domain of Ufd2^13^. In an analogous manner, HUWE1 attaches K48 linkages to unbranched K63-linked chains synthesized by TRAF6 by recognizing K63 linkages through its UIM and UBA domains^14^. Branched K48/K63 chains formed by ITCH and UBR5 are produced through a mechanism that involves the binding of K63-linked chains conjugated by ITCH to the UBA domain of UBR5, a K48-specific E3^15^. Existing evidence suggests that HUWE1, UBR4, and UBR5 may have special roles as chain branching E3s, as they have been demonstrated to collaborate with multiple E3s to form distinct types of branched linkages^11,14,15^.

For branched chains synthesized by individual E3s, the mechanisms of branching vary and are generally less clear. The APC/C acts as a multisubunit scaffold to recruit two different E2s with distinct linkage preferences that work cooperatively to assemble branched K11/K48 chains^10,38^. Interestingly, the APC/C engages UBE2C and UBE2S in different manners to create unique catalytic architectures that promote the different stages of chain initiation and branching^39^. For individual E3s that have the ability to assemble branched polymers with a single E2, mechanisms for building chains that contain at least two different linkages must be intrinsic to the E3, although such mechanisms have yet to be identified. It is notable that the HECT E3s WWP1 and UBE3C, both of which have been shown to form branched chains, contain a non-covalent ubiquitin-binding site within or adjacent to the catalytic HECT domain that could act to facilitate chain branching^32,33^. Alternatively, the topology of the growing chain tethered to the E3-bound substrate could limit the length of an unbranched homotypic chain, thereby indirectly altering the catalytic specificity of the E3 in a way that promotes branching, as suggested for WWP1^32^. The latter model would predict that branching is initiated from the distal ubiquitin on the end of the growing chain, although it is unclear how further branching would be favored over the formation of mixed chains in this model. One possibility is that the chain building activity of the E3 is redirected to internal ubiquitins within the chain, through an as of yet unidentified mechanism, once the growing chain reaches a critical threshold length.

## Physiological functions of branched ubiquitin chains

Recent studies have revealed that branched chains represent a significant fraction of all ubiquitin chains in cells, with current estimates ranging from 5 to 20% depending on the cell type and the method of measurement used^37,40^. The abundance of some types of branched chains is especially high in unstimulated cells. For example, it has been estimated that branched K48/K63 chains make up about 20% of all K63 linkages in U2OS cells^14^. Other types of branched chains are probably of lower abundance but can be synthesized at much higher levels under certain conditions or in response to specific signals. Branched K11/K48 chains, for instance, increase sharply in number during mitosis and in response to proteotoxic stress^10,11,41^, whereas branched K48/K63 chains increase in response to activation of NF-κB signaling^14^. Branched K48/K63 chains also increase greatly in abundance after treatment with proteasome inhibitors, accounting for ~50% of all K63 linkages^14^, suggesting that branched K48/K63 chains are prominent degradation signals in cells. The abundance of other types of branched chains has not been measured directly but is likely to fluctuate in response to context-dependent signals or events, such as bacterial infection in the case of branched K6/K48 chains synthesized by NleL^17,18^.

Not unexpectedly, the first clearly defined function of branched ubiquitin chains was in the degradation of proteins by the proteasome. In a landmark study, the APC/C was found to attach branched K11/K48 chains to mitotic cell cycle regulators, such as cyclin A and Nek2A, targeting them for destruction by the proteasome^10,42^. Branched K11/K48 chains have also been implicated in the degradation of newly synthesized misfolded proteins and cytoplasmic aggregates^11,43^, many of which seem to require the activity of VCP/p97, a AAA + ATPase that prepares ubiquitylated proteins for degradation by the proteasome and binds efficiently to branched K11/K48 chains^10,11,44,45^. Similarly, branched K29/K48 and K48/K63 chains have been shown to target a diverse array of proteins, including substrates of the UFD pathway, ERAD substrates, and apoptotic regulators, for proteasomal degradation^13,15^. Importantly, in many cases, branched chains appear to act as more potent degradation signals than their unbranched counterparts^10,11,13,15^, raising the possibility that branched chains have evolved to promote the disposal of a subset of unwanted or extremely toxic proteins. While the functions of branched chains in degradative pathways have been reviewed in detail elsewhere^45^, we focus here on recent findings pertaining to the role of branched polymers as the preferred signals for proteasomal degradation and highlight the functions of non-degradative branched chains in controlling cell signaling pathways.

The ability of branched chains to act as powerful degradation signals and to carry out non-degradative functions is driven by the specific recognition of branched polymers by effector proteins. These effector proteins harbor ubiquitin-binding domains (UBDs), which recognize a variety of ubiquitylation signals, including branched and unbranched chains, and in many cases show specificity for the types of ubiquitylation marks that they bind to^46,47^. Although there are currently no examples of UBDs that bind exclusively to branched chains, a number of ubiquitin receptors that are directly involved in proteasomal degradation have been shown to bind to branched polymers with higher affinity compared to unbranched chains. Two of the three established ubiquitin receptors of the proteasome, RPN1 and RPN10, bind to branched K11/K48 chains more robustly than their respective unbranched polymers^10,48^. Additionally, VCP/p97, which facilitates proteasomal degradation through multiple mechanisms, including the extraction and unfolding of ubiquitylated substrates^44^, binds more efficiently to branched K11/K48 chains than to unbranched K11, unbranched K48, or mixed K11/K48 chains^10,11^. In yeast, branched K29/48 chains formed by Ufd4 and Ufd2 in the context of the UFD pathway bind more tightly than homotypic K29-linked chains to Rpn10 and the proteasome shuttling factors Rad23 and Dsk2^13^. Interestingly, although K11-linked chains were initially thought to act as prominent degradation signals, recent work has shown that unbranched K11 chains bind only weakly to proteasomes and do not support the degradation of model substrates by proteasomes in vitro^49,50^. Together, these findings support the idea that branched chains are generally more potent degradation signals than their unbranched counterparts.

**Fig. 3:**
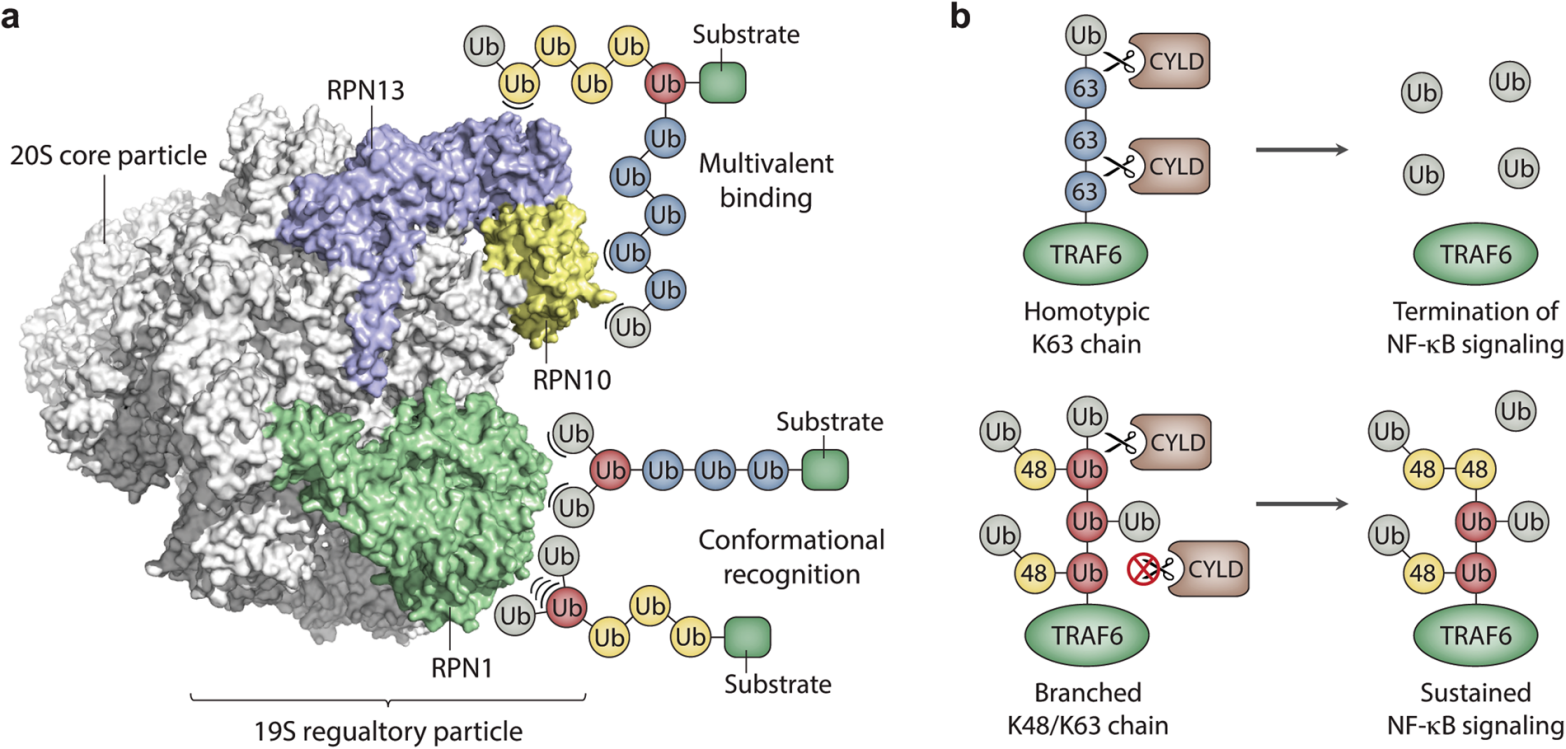
Models for the recognition and functions of branched ubiquitin chains. **a** The binding of branched chains to the proteasome (PDB ID: 5T0J) is illustrated schematically. The ubiquitin-binding subunits of the 19S regulatory particle are colored in blue, yellow, and green; all other proteasome subunits are colored in white. The enhanced binding of branched chains to the proteasome as a result of an increase in the local concentration or “density” of ubiquitin subunits surrounding the substrate is illustrated by the multivalent-binding model. Enhanced binding due to the recognition of novel interaction surfaces created by branching or recognition of the branch point itself is represented by the conformational recognition model. Non-covalent interactions between ubiquitin and proteasome subunits are represented by arcs. The positions of the ubiquitin-binding sites on the proteasome are shown for schematic purposes only. **b** Model for the role of branched K48/K63 chains in the activation of NF-κB signaling. Homotypic K63-linked chains are efficiently disassembled by CYLD, resulting in the removal of K63 linkages from TRAF6 and the termination of NF-κB signaling (top). Branched K48/K63 chains are resistant to CYLD cleavage, resulting in the persistence of K63 linkages on TRAF6 and sustained activation of NF-κB signaling (bottom). Branched ubiquitin subunits modified at both K48 and K63 are colored in red.

How are branched chains recognized so much more efficiently by the proteasome and other components of the degradation machinery? An attractive hypothesis is that branching simply provides an increase in the local concentration or “density” of ubiquitins surrounding the substrate^10,11,42,45^, thus increasing the probability of a productive interaction between the ubiquitylated substrate and its receptor through an avidity effect (Fig. 3a). This hypothesis is supported by studies demonstrating that multiple short chains or monoubiquitin modifications can match or exceed the degradative capacity of one or two longer chains consisting of 4–9 ubiquitins^51–53^. However, the enhanced or specific binding of ubiquitin receptors to recognition sites in the vicinity of the branch point or to unique interaction surfaces created by branching cannot be excluded (Fig. 3a). In fact, a recent study demonstrated that RPN1, which harbors multiple UBDs, binds more robustly to branched K11/K48 triubiquitin compared to K48-linked triubiquitin or mixed-linkage K11/K48 triubiquitin^48^, suggesting that branched K11/K48 chains form a unique structure that is preferentially recognized by RPN1. Enhanced binding to RPN1 may be driven by a hydrophobic interface between the distal ubiquitins of branched K11/K48 triubiquitin that is present only in the branched form of the chain^48^. Clearly, there is still much to be learned about how branched chains are preferentially recognized and processed by the proteasome and other components of the degradation machinery.

In addition to their roles in protein degradation pathways, branched chains can also act as non-degradative signals to control cell signaling events. The best-characterized example of this is in NF-κB signaling, which is regulated by multiple types of ubiquitin chains, including both unbranched K63-linked chains and branched K48/K63 chains^14,21,54,55^. Activation of NF-κB signaling involves the assembly of unbranched K63-linked chains by TRAF6, which in turn bind to TAB2 to activate the TAK1 kinase complex, thus triggering a cascade of downstream events that ultimately leads to the release of active NF-κB and regulation of gene expression. This pathway is antagonized by two deubiquitylating enzymes (DUBs), CYLD and A20, which disassemble K63-linked chains and therefore inhibit the activation of TAK1 and subsequent downstream events. Interestingly, HUWE1 has been shown to add K48-linked ubiquitins to K63-linked chains preassembled by TRAF6, resulting in the formation of branched K48/K63 chains. Branched K48/K63 chains are resistant to cleavage by CYLD, leading to stabilization of K63 linkages and sustained activation of NF-κB signaling (Fig. 3b). Thus, branching can regulate cell signaling by blocking the activity of a DUB and stabilizing ubiquitylation marks that function in a non-degradative context^14^. A similar regulatory mechanism may be involved in the activation of NF-κB signaling by branched M1/K63 chains, which are reported to be resistant to cleavage by A20^20^, although further work is needed to confirm the presence of branched structures in order to validate this model.

## Methods and tools to study branched ubiquitin chains

The detection of branched ubiquitin chains has been more challenging than it has for homotypic chains for several reasons, including low abundance relative to homotypic chains and the existence of inherent limitations of traditional proteomics methods. Ubiquitin mutants carrying various combinations of Lys to Arg mutations that block chain formation through one or more Lys residues have been somewhat useful in the identification of branched chains in vitro^10,13,17,33^. These mutants have been used most successfully in conjunction with in-frame ubiquitin fusion proteins or enzymatically generated substrates modified with ubiquitin chains of defined lengths and linkages^10,13,32^. The use of ubiquitin mutants in cell-based studies has been less fruitful due to a number of confounding factors, including the presence of wild-type endogenous ubiquitin, off-target effects caused by global perturbation of ubiquitin dynamics, and the presence of DUBs and protein degradation pathways. One approach based on a ubiquitin mutant that has been used with success in the detection of branched chains in cells is the TEV cleavage method, which involves the expression of a ubiquitin variant containing an engineered TEV protease cleavage site after Gly53 or Glu64 of ubiquitin. In this method, affinity purification of ubiquitylated substrates from cells expressing the TEV ubiquitin mutant is followed by TEV cleavage and western blotting to detect FLAG-reactive peptides that are diagnostic of chain branching^10,13^.

**Fig. 4:**
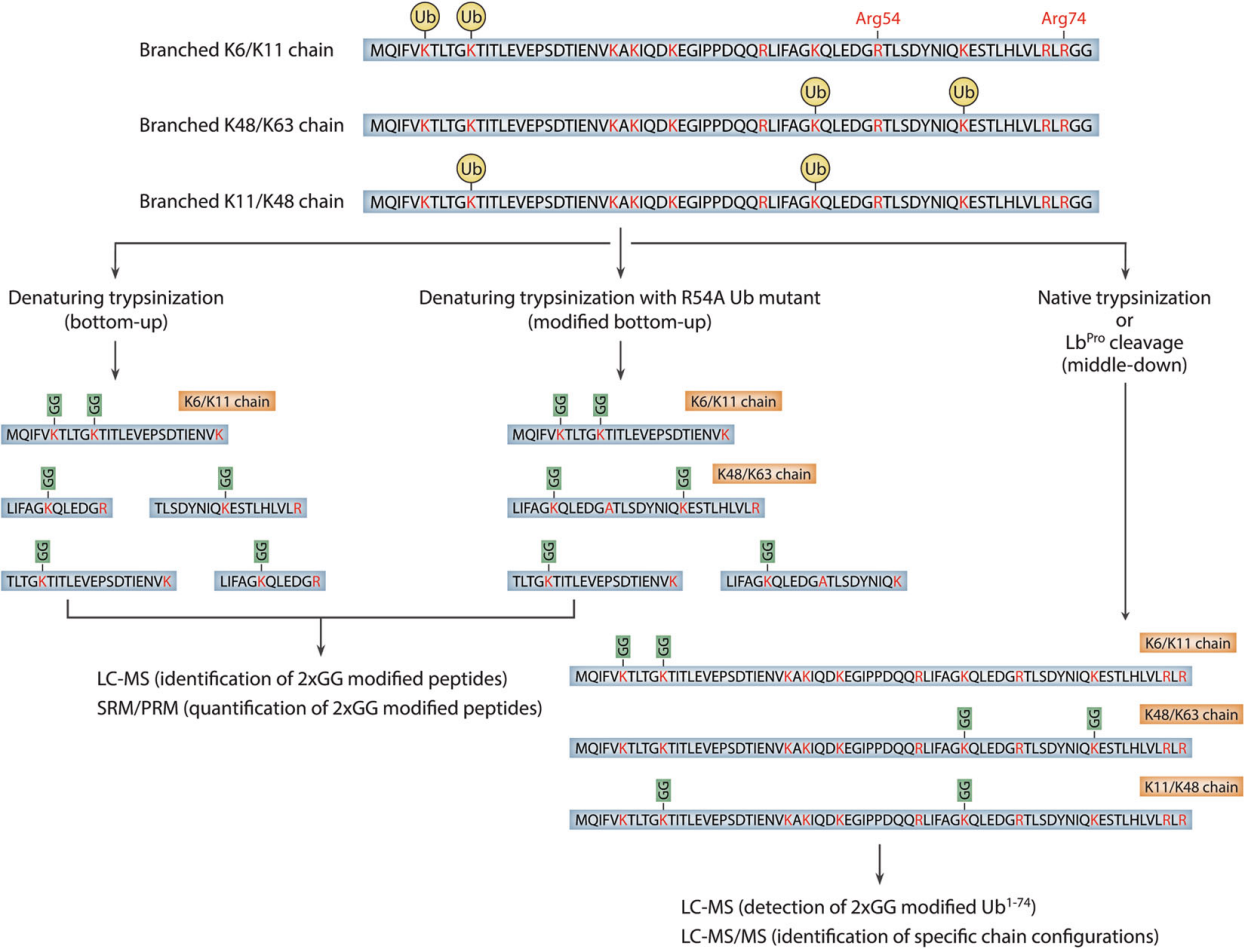
Mass spectrometry-based workflows to detect branched ubiquitin chains. Three different approaches to detect branched chains are illustrated. In the classical bottom-up approach, branched chains involving neighboring lysines can be detected, but all other types of branched chains are invisible due to the cleavage of branched peptides at intervening lysines or arginines (left). Mutation of the single arginine located between K48 and K63 of ubiquitin (Arg54) allows the detection of branched K48/K63 chains using classical bottom-up methods (middle). In principle, other types of branched chains can be detected in this manner. In the middle-down approach, ubiquitin chains are digested with trypsin under native conditions or cleaved after Arg74 with a site-specific protease (right). This approach leaves all possible combinations of branch points intact. Specific chain configurations can then be identified by tandem mass spectrometry. LC–MS liquid chromatography-mass spectrometry, SRM selected reaction monitoring, PRM parallel reaction monitoring, LC–MS/MS liquid chromatography-tandem mass spectrometry.

While the aforementioned methods have been instrumental in the initial discovery of branched chains, they are limited because they generally provide only qualitative information and are prone to artifacts due to altered conjugation properties of the ubiquitin mutants. The gold standard for detecting branched chains has accordingly been mass spectrometry, and a number of recent advances in proteomic technologies have facilitated their study. Traditional bottom-up approaches, which rely on the digestion of ubiquitylated proteins with trypsin and a resulting 114 Da Gly–Gly remnant on the modified Lys residue, have been used extensively to detect both homotypic chains and branched chains involving neighboring Lys residues, as is the case for K6/K11, K27/K29, and K29/K33 polymers^16,36,56–58^. However, the detection of branched chains involving non-neighboring Lys residues or Lys residues separated by one or more arginines is not possible using traditional bottom-up approaches, due to cleavage and the resulting loss of the branched peptide (Fig. 4). The use of a ubiquitin mutant lacking the single Arg residue found between K48 and K63 of ubiquitin (R54A) has enabled the detection of branched K48/K63 chains using bottom-up methods^14,15^. However, an extensive number of point mutations would be needed to detect other branched peptides in this manner, and the increased size of these peptides would likely present challenges to their detection^59,60^.

Middle-down mass spectrometry approaches, which involve the minimal digestion of ubiquitin chains under native conditions to cleave specifically after Arg74, have so far proven to be the most universally applicable in the detection of branched chains. In the middle-down workflow, the ubiquitin polypeptide is left largely intact after cleavage of ubiquitin chains, and multiple Gly–Gly modifications are detected concurrently on a single polypeptide (Fig. 4). This approach has been used to estimate the abundance of branched chains in cells and to detect specific chain configurations, including those comprised of branched K6/K48, K11/K48, and K29/K48 linkages^18,37,40,41^. In a recent study, a middle-down approach based on the activity of an engineered viral protease (Lb^Pro^) with high specificity for cleavage after Arg74 of ubiquitin was used to detect branched chains in vitro and in cellulo. This method, dubbed Ub-clipping, enabled the quantification of branched chains in cells and led to the surprising conclusion that as much as 20% of polymerized ubiquitin exists in a branched form^37^. It should be noted that strategies for detecting branched chains by mass spectrometry often involve an enrichment step to increase the recovery of polyubiquitylated proteins prior to enzymatic cleavage. In recent studies, tandem ubiquitin-binding entities (TUBEs) and linkage-specific antibodies have both been used effectively towards this goal^37,41^. Enrichment is necessary to facilitate the detection of branched chains because of the large amount of free ubiquitin and monoubiquitylated proteins present in cell extracts that can interfere with the detection of branched polymers, especially those that are present at low levels.

Other methods that have contributed to the study of branched chains include the use of a bispecific antibody that recognizes branched K11/K48 chains and linkage-specific DUBs to determine higher-order chain architecture. It is worth noting that the bispecific antibody for branched K11/K48 chains also recognizes mixed K11/K48 chains and has a lower affinity for homotypic K11 and K48 linkages^10^. Thus, the detection of branched K11/K48 chains must be confirmed by other methods in order to substantiate claims of branching. A technique based on the linkage-specific properties of DUBs, known as Ubiquitin Chain Restriction, has been used in several cases to establish the general architecture of branched chains^61^. For example, the K63-specific AMSH and K48-specific OTUB1 DUBs were used to show that the formation of branched K48/K63 chains by TRAF6 and HUWE1 during NF-kB signaling involves the assembly of K48 linkages on preformed K63-linked chains^14^. Finally, the recent use of click chemistry to synthesize non-hydrolyzable versions of branched K6/K11, K11/K48, and K48/K63 chains, which are resistant to cleavage by DUBs, should prove to be valuable in the identification of novel ubiquitin-binding receptors for branched chains^62^. It is noteworthy that non-hydrolyzable versions of homotypic chains have already been used with success to identify previously unknown interactors of atypical unbranched chains^63^.

## Concluding statements and future perspectives

While the architectures and functions of branched ubiquitin chains have clearly started to emerge in recent years, there are still many remaining questions about how these signals are produced, how they are recognized, how they impact cell physiology, and how they differ from their more primitive homotypic relatives (Box 1). Branched chains with unique architectures are likely to be discovered in the future, and the identification of additional chain branching E3s will contribute to our understanding of how these signals are encoded by the ubiquitylation machinery. HECT E3s are good candidates for chain branching enzymes, as a number of these ligases have been reported to synthesize heterotypic chains with mixed linkages^32,33,64–68^. There is also much left to be learned about the structures of branched chains that are already known to exist and have been assigned to essential cellular functions. Several recently reported atomic structures of branched K11/K48 triubiquitin have provided some clues about how the spatial landscapes of branched chains might differ from those of their unbranched counterparts^48^. However, the structures of other types of branched polymers consisting of differing lengths, linkages, and branch point locations are currently unknown.

Despite recent progress, the specific functions of branched ubiquitin chains, which are mediated by effector proteins that recognize these signals, are still only in the early stages of their discovery. While the functions of branched K11/K48, K29/K48, and K48/K63 chains are understood in some detail, the physiological roles of other branched polymers have not yet been identified. Existing evidence suggests critical biological functions for branched M1/K63, K6/K48, and K11/K63 chains^17,19–23,69^, although further research is needed to establish clear structure/function relationships for these polymers. Additional functions, both proteolytic and non-proteolytic, are likely to be discovered for branched chains that are already understood in some detail. In fact, branched K11/K48 chains have recently been implicated in the regulation of histone stability to control gene expression and in the degradation-independent activation of the Met4 transcription factor^12,70^. Interestingly, branched K11/K48 chains are conjugated to histone proteins at specific chromatin locations during mitosis to ensure their degradation and the expression of genes that maintain stem cell identity in the ensuing cell cycle. In this newly discovered pathway, APC/C is recruited to the promoters of pluripotency genes by WDR5, a chromatin-associated factor that is thought to function as an APC/C adaptor to promote the formation of branched K11/K48 chains on histones^12^. Although the role of branched K11/K48 chains in Met4 activation is less clear, existing evidence suggests a model in which heterotypic K11/K48 chains disrupt an autoinhibitory interaction between the tandem UBDs of Met4 and covalently attached homotypic K48-linked chains^70^.

Clearly, new methods and technological advances that extend the capabilities of those described in this review article will be needed to fully uncover the structures and functions of branched chains. The rationale for investing in these technologies is considerable because of the direct link that these signals have to human health and disease. Branched polymers have been implicated in the degradation of aggregation-prone proteins that are directly responsible for causing neurodegenerative diseases^11^, and Parkin, a ubiquitin ligase that is often mutated in early-onset Parkinson’s disease, was recently reported to synthesize branched chains^34,37^. Ultimately, the “ubiquitin code” will need to be expanded to include branched polymers, and cracking this expanded code will undoubtedly be a major challenge for the field. An additional layer of complexity must also be accounted for because it is now well documented that free ubiquitin and ubiquitin chains can both be post-translationally modified by a number of small chemical groups, including phosphoryl, acetyl, ADP-ribosyl, and phosphoribosyl groups^71–73^. Accordingly, it is perhaps not too far-fetched to think of ubiquitylation marks in a manner that is analogous to histone modifications, with, in this case, the combinatorial effects of multiple chain linkages, branch points, and chemical group additions, determining the ultimate fate of a protein that a particular ubiquitylation mark is attached to.

Box 1 Unanswered questions

- How is the formation of branched chains regulated? What are the molecular events that promote or inhibit the assembly of branched polymers? Possible mechanisms include the context-dependent recruitment of chain branching E2s or E3s and regulation through post-translational modifications of ubiquitin chains that stimulate or block chain branching.
- What roles do DUBs have in editing the higher-order structures of branched chains? The architectures of some branched chains may be determined by the combined activities of chain branching E2/E3 enzymes and DUBs that cleave specific linkages.
- What are the molecular “rules” that govern the formation of branched chains? Are there particular acceptor sites in proteins that are preferred for the addition of branched chains? How many ubiquitin subunits do branched chains typically contain? Is there a preference for initiating branch points at proximal, internal, or distally located ubiquitins within a chain?
- Are there novel ubiquitin receptors and UBDs that bind specifically to branched chains? If so, what are the molecular mechanisms of chain recognition? Are the number and location of branch points important for the recognition of branched polymers?
- What are the architectures and physiological functions of branched chains that have not yet been characterized in detail? Existing evidence suggests critical functions for branched M1/K63, K6/K48, and K11/K63 chains, but further work is needed to establish their general architectures and roles in cell physiology. Additional types of branched chains (i.e., K6/K11, K27/K29, K29/K33) have been documented but currently have no known functions.
- What is the evolutionary relationship between homotypic polymers and branched chains? Have branched chains evolved in higher eukaryotic organisms to fulfill specific roles, such as the rapid disposal of highly toxic protein aggregates?

## References

[1] Rape M (2018). Ubiquitylation at the crossroads of development and disease. Nat. Rev. Mol. Cell Biol..

[2] Pohl C, Dikic I (2019). Cellular quality control by the ubiquitin-proteasome system and autophagy. Science.

[3] Oh E, Akopian D, Rape M (2018). Principles of ubiquitin-dependent signaling. Annu. Rev. Cell Dev. Biol..

[4] Schwertman P, Bekker-Jensen S, Mailand N (2016). Regulation of DNA double-strand break repair by ubiquitin and ubiquitin-like modifiers. Nat. Rev. Mol. Cell Biol..

[5] Foot N, Henshall T, Kumar S (2017). Ubiquitination and the regulation of membrane proteins. Physiol. Rev..

[6] Komander D, Rape M (2012). The ubiquitin code. Annu. Rev. Biochem..

[7] Akutsu M, Dikic I, Bremm A (2016). Ubiquitin chain diversity at a glance. J. Cell Sci..

[8] Swatek KN, Komander D (2016). Ubiquitin modifications. Cell Res..

[9] Grumati P, Dikic I (2018). Ubiquitin signaling and autophagy. J. Biol. Chem..

[10] Meyer HJ, Rape M (2014). Enhanced protein degradation by branched ubiquitin chains. Cell.

[11] Yau RG (2017). Assembly and function of heterotypic ubiquitin chains in cell-cycle and protein quality control. Cell.

[12] Oh E (2020). Gene expression and cell identity controlled by anaphase-promoting complex. Nature.

[13] Liu C, Liu W, Ye Y, Li W (2017). Ufd2p synthesizes branched ubiquitin chains to promote the degradation of substrates modified with atypical chains. Nat. Commun..

[14] Ohtake F, Saeki Y, Ishido S, Kanno J, Tanaka K (2016). The K48-K63 branched ubiquitin chain regulates NF-κB signaling. Mol. Cell.

[15] Ohtake F, Tsuchiya H, Saeki Y, Tanaka K (2018). K63 ubiquitylation triggers proteasomal degradation by seeding branched ubiquitin chains. Proc. Natl Acad. Sci. USA.

[16] Kim HT (2007). Certain pairs of ubiquitin-conjugating enzymes (E2s) and ubiquitin-protein ligases (E3s) synthesize nondegradable forked ubiquitin chains containing all possible isopeptide linkages. J. Biol. Chem..

[17] Hospenthal MK, Freund SM, Komander D (2013). Assembly, analysis and architecture of atypical ubiquitin chains. Nat. Struct. Mol. Biol..

[18] Valkevich EM, Sanchez NA, Ge Y, Strieter ER (2014). Middle-down mass spectrometry enables characterization of branched ubiquitin chains. Biochemistry.

[19] Emmerich CH (2013). Activation of the canonical IKK complex by K63/M1-linked hybrid ubiquitin chains. Proc. Natl Acad. Sci. USA.

[20] Wertz IE (2015). Phosphorylation and linear ubiquitin direct A20 inhibition of inflammation. Nature.

[21] Cohen P, Strickson S (2017). The role of hybrid ubiquitin chains in the MyD88 and other innate immune signalling pathways. Cell Death Differ..

[22] Boname JM (2010). Efficient internalization of MHC I requires lysine-11 and lysine-63 mixed linkage polyubiquitin chains. Traffic.

[23] Goto E (2010). Contribution of lysine 11-linked ubiquitination to MIR2-mediated major histocompatibility complex class I internalization. J. Biol. Chem..

[24] Pickart CM, Eddins MJ (2004). Ubiquitin: structures, functions, mechanisms. Biochim. Biophys. Acta.

[25] Neutzner M, Neutzner A (2012). Enzymes of ubiquitination and deubiquitination. Essays Biochem..

[26] Metzger MB, Pruneda JN, Klevit RE, Weissman AM (2014). Ring-type E3 ligases: master manipulators of E2 ubiquitin-conjugating enzymes and ubiquitination. Biochim. Biophys. Acta.

[27] Lorenz S (2018). Structural mechanisms of HECT ubiquitin ligases. Biol. Chem..

[28] Walden H, Rittinger K (2018). RBR ligase-mediated ubiquitin transfer: a tale with many twists and turns. Nat. Struct. Mol. Biol..

[29] Pao KC (2018). Activity-based E3 ligase profiling uncovers an E3 ligase with esterification activity. Nature.

[30] Deol KK, Lorenz S, Strieter ER (2019). Enzymatic logic of ubiquitin chain assembly. Front. Physiol..

[31] Kirkpatrick DS (2006). Quantitative analysis of in vitro ubiquitinated cyclin B1 reveals complex chain topology. Nat. Cell Biol..

[32] French ME (2017). Mechanism of ubiquitin chain synthesis employed by a HECT domain ubiquitin ligase. J. Biol. Chem..

[33] Wang M, Cheng D, Peng J, Pickart CM (2006). Molecular determinants of polyubiquitin linkage selection by an HECT ubiquitin ligase. EMBO J..

[34] Deol KK, Eyles SJ, Strieter ER (2020). Quantitative middle-down MS analysis of Parkin-mediated ubiquitin chain assembly. J. Am. Soc. Mass Spectrom..

[35] Durcan TM (2014). USP8 regulates mitophagy by removing K6-linked ubiquitin conjugates from Parkin. EMBO J..

[36] Ordureau A (2014). Quantitative proteomics reveal a feedforward mechanism for mitochondrial PARKIN translocation and ubiquitin chain synthesis. Mol. Cell.

[37] Swatek KN (2019). Insights into ubiquitin chain architecture using Ub-clipping. Nature.

[38] Wickliffe KE, Williamson A, Meyer HJ, Kelly A, Rape M (2011). K11-linked ubiquitin chains as novel regulators of cell division. Trends Cell Biol..

[39] Brown NG (2016). Dual RING E3 architectures regulate multiubiquitination and ubiquitin chain elongation by APC/C. Cell.

[40] Crowe SO, Rana A, Deol KK, Ge Y, Strieter ER (2017). Ubiquitin chain enrichment middle-down mass spectrometry enables characterization of branched ubiquitin chains in cellulo. Anal. Chem..

[41] Rana A, Ge Y, Strieter ER (2017). Ubiquitin chain enrichment middle-down mass spectrometry (ubichem-ms) reveals cell-cycle dependent formation of Lys11/Lys48 branched ubiquitin chains. J. Proteome Res..

[42] Lopez-Mosqueda J, Dikic I (2014). Deciphering functions of branched ubiquitin chains. Cell.

[43] Samant RS, Livingston CM, Sontag EM, Frydman J (2018). Distinct proteostasis circuits cooperate in nuclear and cytoplasmic protein quality control. Nature.

[44] van den Boom J, Meyer H (2018). VCP/p97-mediated unfolding as a principle in protein homeostasis and signaling. Mol. Cell.

[45] Haakonsen DL, Rape M (2019). Branching out: improved signaling by heterotypic ubiquitin chains. Trends Cell Biol..

[46] Husnjak K, Dikic I (2012). Ubiquitin-binding proteins: decoders of ubiquitin-mediated cellular functions. Annu. Rev. Biochem..

[47] Randles L, Walters KJ (2012). Ubiquitin and its binding domains. Front. Biosci..

[48] Boughton AJ, Krueger S, Fushman D (2020). Branching via K11 and K48 bestows ubiquitin chains with a unique interdomain interface and enhanced affinity for proteasomal subunit Rpn1. Structure.

[49] Grice GL (2015). The proteasome distinguishes between heterotypic and homotypic lysine-11-linked polyubiquitin chains. Cell Rep..

[50] Martinez-Fonts K (2020). The proteasome 19S cap and its ubiquitin receptors provide a versatile recognition platform for substrates. Nat. Commun..

[51] Lee BH (2016). USP14 deubiquitinates proteasome-bound substrates that are ubiquitinated at multiple sites. Nature.

[52] Lu Y, Lee BH, King RW, Finley D, Kirschner MW (2015). Substrate degradation by the proteasome: a single-molecule kinetic analysis. Science.

[53] Braten O (2016). Numerous proteins with unique characteristics are degraded by the 26S proteasome following monoubiquitination. Proc. Natl Acad. Sci. USA.

[54] Chen J, Chen ZJ (2013). Regulation of NF-κB by ubiquitination. Curr. Opin. Immunol..

[55] Iwai K (2014). Diverse roles of the ubiquitin system in NF-κB activation. Biochim. Biophys. Acta.

[56] Peng J (2003). A proteomics approach to understanding protein ubiquitination. Nat. Biotechnol..

[57] Xu P (2009). Quantitative proteomics reveals the function of unconventional ubiquitin chains in proteasomal degradation. Cell.

[58] Phu L (2011). Improved quantitative mass spectrometry methods for characterizing complex ubiquitin signals. Mol. Cell. Proteom..

[59] Fricker LD (2015). Limitations of mass spectrometry-based peptidomic approaches. J. Am. Soc. Mass Spectrom..

[60] Kim MS, Zhong J, Pandey A (2016). Common errors in mass spectrometry-based analysis of post-translational modifications. Proteomics.

[61] Hospenthal MK, Mevissen TET, Komander D (2015). Deubiquitinase-based analysis of ubiquitin chain architecture using Ubiquitin Chain Restriction (UbiCRest). Nat. Protoc..

[62] Zhao X, Scheffner M, Marx A (2019). Assembly of branched ubiquitin oligomers by click chemistry. Chem. Commun..

[63] Zhang X (2017). An interaction landscape of ubiquitin signaling. Mol. Cell.

[64] Sheng Y (2012). A human ubiquitin conjugating enzyme (E2)-HECT E3 ligase structure-function screen. Mol. Cell. Proteom..

[65] Michel MA (2015). Assembly and specific recognition of K29- and K33-linked polyubiquitin. Mol. Cell.

[66] Fang NN, Zhu M, Rose A, Wu KP, Mayor T (2016). Deubiquitinase activity is required for the proteasomal degradation of misfolded cytosolic proteins upon heat-stress. Nat. Commun..

[67] Michel MA, Swatek KN, Hospenthal MK, Komander D (2017). Ubiquitin linkage-specific affimers reveal insights into K6-linked ubiquitin signaling. Mol. Cell.

[68] Singh S, Ng J, Nayak D, Sivaraman J (2019). Structural insights into a HECT-type E3 ligase AREL1 and its ubiquitination activities in vitro. J. Biol. Chem..

[69] Lin DY, Diao J, Zhou D, Chen J (2011). Biochemical and structural studies of a HECT-like ubiquitin ligase from *Escherichia coli* O157:H7. J. Biol. Chem..

[70] Li Y (2019). Proteomics links ubiquitin chain topology change to transcription factor activation. Mol. Cell.

[71] Kliza K, Husnjak K (2020). Resolving the complexity of ubiquitin networks. Front. Mol. Biosci..

[72] Ohtake F, Tsuchiya H (2017). The emerging complexity of ubiquitin architecture. J. Biochem..

[73] Song L, Luo ZQ (2019). Post-translational regulation of ubiquitin signaling. J. Cell Biol..

